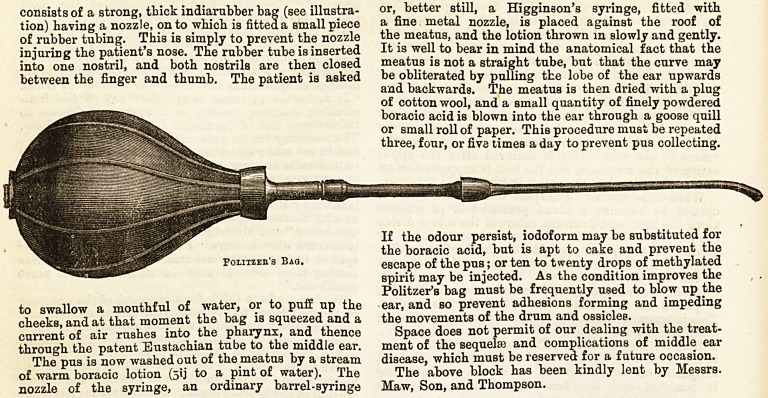# The Treatment of Chronic Suppuration of the Middle Ear

**Published:** 1892-11-05

**Authors:** 


					ROYAL INFIRMARY, EDINBURGH.
The Treatment of Chronic Suppuration of
the Middle Ear.
There may be some of the commoner surgical
affections regarding which it would be possible to
exaggerate the importance of prompt, patient and per-
sistent treatment. Chronic suppuration of the middle
ear is not one of these. The management of this com-
paratively frequent malady is one of the most
responsible duties a medical man can undertake.
Unfortunately the danger of the condition is not
appreciated by the laity, amongst whom a belief exists
that the discharge from the ear has a salutary effect on
the patient, a belief which, like most popular ideas on
medical subjects, has in it a certain amount of truth.
" It is better to come away," they say of the foul-
smelling discharge which is a source of disgust to
themselves, and of imminent danger to the sufferer.
The necessity for its presence is thus tacitly admitted,
and in too many cases the medical^ attendant neglects
to undeceive the ignorant, finding in this vulgar error
an easy means of escaping from a duty at once dis-
agreeable, tedious and difficult. The sarcastic remark
is made by Roosa that " the Creator would have made
us with discharging ears if they were necessary for our
well-being," and McBride denounces those to whom it
is directed when he says, " I hesitate to apply the proper
epithet to such empirical statements and prophecies
coming from medical men in whom patients place
confidence."
We have only to think for a moment of the anatomy
of the middle ear to appreciate the almost endless mis-
fortunes which may befall the unhappy patient in whom
it is the seat of chronic suppuration. The minute
cavity, measuring in the adult less than half an inch
in every diameter, for the most part bounded by un-
yielding bony walls, contains the delicate mechanical
portion of the hearing organ. The tympanic membrane
or drum of the ear forms its outer wall, and is more or
less damaged in every case. It is shut off by a very
thin shell of bone from the dura mater and tempero-
sphenoidal lobe of the brain above, and from the
jugular vein below. The fact that the mastoid cells lie
close behind and communicate freely with the tympanic
cavity is of importance from the point of view of treat-
ment, and the close proximity of the lateral sinus adds
a source of danger when the pus reaches the air cells.
Lastly, passing through one of the walls runs the
facial nerve, with a nerve of taste?the chorda tympanu
Add to all this that the cavity, when the seat of sup-
puration, communicates freely with the external air
and its inevitable septic organisms, and the possibilities
for evil are evident.
The pent-up septic pus clogs and impedes the move-
ments of the auditory ossicles, ultimately leading to
ankylosis or necrosis with resulting deafness. Spreading
into the mastoid cells, it sets up inflammation, abscess,
or even necrosis of bone there. Phlebitis and embolus
of the lateral sinus and jugular vein is not uncommon,
and abscess in the tempero-sphenoidal lobe of the
brain, or in the cerebellum are by no means rare. Of
less import, but not therefore trivial, is the facial
paralysis and loss of taste associated with damage to
nerves.
The gravity of these conditions is sufficient to em-
phasise the importance of treating thoroughly an
affection which may give rise to any or all of
them.
As the suppuration in a majority of cases follows
on one or other of the exanthemata, e.g., scarlet fever or
measles, the preventive treatment consists in keeping
a sharp look out in all cases of these diseases, and by
early incision of the membrane, preventing its exten-
sive destruction, and evacuating the pus. The condi-
tion is prevented becoming chronic by improving the
general health of the patient by tonics, good food,,
fresh air, &c., and frequent syringing of the ear.
When once established, the aim of our treatment is
to establish a condition of aseptic cleanliness.
If the pus be thick and caked in the meatus it is
readily softened by pouring into the ear a small quantity
of warm water, in which a pinch of bicarbonate of soda
(gr. v. to 3 i.) has been dissolved.
By the aid of Politzer's bag the pus in the middle
ear may be blown into the meatus. This apparatus
&2 THE HOSPITAL. Nov. 5, 1892.
consists of a strong, thick indiarubber bag (see illustra-
tion) having a nozzle, on to which is fitted a small piece
of rubber tubing. This is simply to prevent the nozzle
injuring the patient's nose. The rubber tube is inserted
into one nostril, and both nostrils are then closed
between the finger and thumb. The patient is asked
to swallow a mouthful of water, or to puff up the
cheeks, and at that moment the bag is squeezed and a
current of air rushes into the pharynx, and thence
through the patent Eustachian tube to the middle ear.
The pus is now washed out of the meatus by a stream
of warm boracic lotion (5ij to a pint of water). The
nozzle of the syringe, an ordinary barrel-syringe
or, better still, a Higginson's syringe, fitted with,
a fine metal nozzle, is placed against the roof of
the meatus, and the lotion thrown in slowly and gently.
It is well to bear in mind the anatomical fact that the
meatus is not a straight tube, but that the curve may
be obliterated by pulling the lobe of the ear upwards
and backwards. The meatus is then dried with a plug
of cotton wool, and a small quantity of finely powdered
boracic acid is blown into the ear through a goose quill
or small roll of paper. This procedure must be repeated
three, four, or five times a day to prevent pus collecting.
If the odour persist, iodoform may be substituted for
the boracic acid, but is apt to cake and prevent the
escape of the pus; or ten to twenty drops of methylated
spirit may be injected. As the condition improves the
Politzer's bag must be frequently used to blow up the
ear, and bo prevent actnesions lorming ana impeding
the movements of the drum and ossicles.
Space does not permit of our dealing with the treat-
ment of the sequelae and complications of middle ear
disease, which must be reserved for a future occasion.
The above block has been kindly lent by Messrs.
Maw, Son, and Thompson.
consists of a strong, thick indiarabber bag (see illustra-
tion) having a nozzle, on to which is fitted a small piece
of rubber tubing. This is simply to prevent the nozzle
injuring the patient's nose. The rubber tube is inserted
into one nostril, and both nostrils are then closed
between the finger and thumb. The patient is asked
or, better still, a Higginson's syringe, fitted with
a fine metal nozzle, is placed against the roof of
the meatus, and the lotion thrown in slowly and gently.
It is well to bear in mind the anatomical fact that the
meatus is not a straight tube, but that the curve may
be obliterated by pulling the lobe of the ear upwards
and backwards. The meatus is then dried with a plug
of cotton wool, and a small quantity of finely powdered
boracic acid is blown into the ear through a goose quill
or small roll of paper. This procedure must be repeated
three, four, or five times a day to prevent pus collecting.
If the odour persist, iodoform may be substituted for
the boracic acid, but is apt to cake and prevent the
escape of the pus; or ten to twenty drops of methylated
spirit may be injected. As the condition improves the
Politzer's bag must be frequently used to blow up the
ear, and so prevent adhesions forming and impeding
the movements of the drum and ossicles.
Space does not permit of our dealing with the treat-
ment of the sequelae and complications of middle ear
disease, which must be reserved for a future occasion.
The above block has been kindly lent by Messrs.
Maw, Son, and Thompson.
to swallow a mouthful of water, or to puff up the
cheeks, and at that moment the bag is squeezed and a
current of air rushes into the pharynx, and thence
through the patent Eustachian tube to the middle ear.
The pus is now washed out of the meatus by a stream
of warm boracic lotion (5ij to a pint of water). The
nozzle of the syringe, an ordinary barrel-syringe
Foliizeh's Bag.

				

## Figures and Tables

**Figure f1:**